# Effect of compound polysaccharide on immunity, antioxidant capacity, gut microbiota, and serum metabolome in kittens

**DOI:** 10.3389/fmicb.2025.1500961

**Published:** 2025-03-05

**Authors:** Yixuan Xie, Shiyan Jian, Limeng Zhang, Baichuan Deng

**Affiliations:** ^1^College of Animal Science, South China Agricultural University, Guangzhou, China; ^2^Guangzhou Qingke Biotechnology Co., Ltd., Guangzhou, Guangdong, China

**Keywords:** *Astragalus* polysaccharide, immunity, kitten, pet food, *Poria cocos* polysaccharide, microbiota, metabolomics

## Abstract

**Introduction:**

This study was conducted to investigate the effects of compound polysaccharides (CP), composed of *Astragalus* polysaccharide and *Poria cocos* polysaccharide, on immunity, antioxidant capacity, gut microbiota, and serum metabolome in kittens.

**Methods:**

A total of 14 4-month-old kittens, with an average body weight of 2.39 kg, were used in a 56-day experiment. They were randomly assigned to the control (CON) group (*n* = 7) and CP group (*n* = 7). Blood samples and fresh feces were collected at the end of the experimental period.

**Results:**

The results displayed that supplementation with CP increased the concentrations of serum immunoglobulin A, immunoglobulin G, interleukin 6, and tumor necrosis factor-*α* (*p <* 0.05). However, there was no difference in the concentrations of serum amyloid A between the two groups (*p* > 0.05). Furthermore, the serum biochemical parameters of all the kittens were within the reference range. The relative abundance of beneficial bacteria (*norank_f__Butyricicoccaceae* and *Bacteroides plebeius*) was higher in the CP group (*p* < 0.05), while the opportunistic pathogen (*Anaerotruncus*) was lower in the CP group (*p* < 0.05). In addition, serum metabolomic analysis demonstrated that the differential metabolites, including arachidonic acid, dihomo-gamma-linolenic acid, and glycine, and the relevant metabolic pathway, including glyoxylate and dicarboxylate metabolism, glycine, serine, and threonine metabolism, and biosynthesis of unsaturated fatty acids, were implicated in regulating immune function in the kitten after CP treatment.

**Conclusion:**

CP supplementation can enhance immune function in kittens and increase the relative abundance of beneficial gut microbiota, and does not lead to generalized inflammation. Dietary supplementation with CP may generate nutritional benefits in kittens, and this study offers insight into the development of functional pet food for kittens.

## 1 Introduction

Newborn kittens rely on maternal antibodies in the colostrum to guarantee an adequate serum immunoglobulin level, which is essential for proper immune response (Rossi et al., 2021). A previous study has indicated that the maternal antibody titers in cats decrease between 4 and 14 weeks after delivery, and weaning is a challenging stage for neonatal mammals (Lei Liu et al., 2020; Pereira et al., 2023). Kittens are susceptible to diseases due to reduced maternal antibodies, immature immune systems, separation from their mother, and relocation during the weaning period (Gore et al., 2021). Nutrition plays a vital role in the health of weaning kittens by meeting their nutritional requirements for the immune system and organ development, with long-term effects on overall health (Rossi et al., 2021). The most important impact of nutrition occurs during the development of their immune system (Cunningham-Rundles et al., 2005). The gut microbiota plays an important role in gut health and immune system development (Jia et al., 2011; Mengxia Wang et al., 2013). In addition, the composition of gut microbiota in kittens is modulated by diets and the weaning period process (Hooda et al., 2013; Jia et al., 2011). Therefore, the post-weaning period is when optimal nutrition can help support the immune system.

Plant polysaccharides, serving as functional ingredients, have attracted much attention due to their natural, non-toxic properties and multiple biological effects in recent years (Zhang et al., 2023). *Astragalus* polysaccharide (APS) is an important active component extracted from dried roots of *Astragalus*, known as “Huangqi” in China (Xu et al., 2023). APS is a kind of water-soluble heteropolysaccharide, mainly composed of nine ratios of monosaccharides, namely, glucose, galactose, arabinose, rhamnose, mannose, xylose, fucose, fructose, and ribose (Han et al., 2019; Wang et al., 2023). A large number of *in vivo* and *in vitro* studies have demonstrated the immunomodulatory, antioxidant, and antitumor effects of APS (Huang et al., 2017; Li C. et al., 2022; Li et al., 2009; Zhao et al., 2022). In addition, researchers have confirmed that APS can enhance the abundance and diversity of intestinal microbiota (Yang et al., 2022; Zhao W. et al., 2023). Similarly, *Poria cocos* polysaccharide (PP), derived from *Poria cocos,* comprises ribose, arabinose, xylose, mannose, glucose, and galactose. There are two kinds of polysaccharides in the sclerotium of *Poria cocos*: water-soluble polysaccharides and alkali-soluble polysaccharides. The main active components are water-soluble polysaccharides (Tan et al., 2023; Wang D. et al., 2020; Wang K. et al., 2020). PP exhibits various biological activities, such as antioxidation, immunomodulation, antidepression, and antitumor functions (Khan et al., 2018; Lai et al., 2022; Zhang et al., 2018). However, there is a lack of studies on the effect of APS or PP supplementation on kittens. Thus, this study aimed to investigate the effect of compound polysaccharide (CP) on immunity, antioxidant capacity, gut microbiota, and serum metabolome in kittens. CP was composed of APS and PP, and the experimental diet was supplemented with 0.1% APS and 0.2% PP. We hypothesized that dietary supplementation with CP may enhance the immunity of weaning kittens as maternal antibody titers decrease and endogenous antibody production is activated during this period.

## 2 Materials and methods

### 2.1 Polysaccharides

APS and PP were purchased from Xi’an Jinshuo Fruit Industry Co., Ltd. APS was extracted from the roots of *Astragalus,* and PP was extracted from *Poria cocos.* CP was composed of APS and PP, and the experimental diet was supplemented with 0.1% APS and 0.2% PP.

### 2.2 Animal and housing

A total of 14 healthy weaning shorthair kittens (10 males and 4 females) from 4 litters, with a mean body weight (BW) of 2.39 ± 0.78 kg and aged 4 months, were included in this study. The kittens were weaned at 3 months of age and then fed the basal diet for 1 month. During the months 3–4, the kittens remained with their litters and in the rooms they were born. At the end of the pre-test period, kittens aged 4 months received the veterinary examination and were relocated from the nursery to another laboratory room (these were considered mildly challenging events). After relocation, the kittens were individually housed at the laboratory in Qingke Biotechnology Co., Ltd. (Guangzhou, China) under controlled temperature and light conditions (12-h light/dark cycle, temperature 23.5 ± 1°C, relative humidity 60 ± 10%) during the test period. Each housing cage (1.1 m × 0.7 m × 0.7 m) had separate areas for feeding, defecation, and resting. The kittens were allowed to socialize with each other and play outside the cages during the non-feeding time. Furthermore, the kittens can access various toys and interact with humans during the day. The cages and rooms were cleaned and sanitized daily. All kittens were dewormed and vaccinated 1 month before the experiment. No drugs, such as antibiotics, were administered during the experiment. Kittens had free access to fresh water throughout the study.

### 2.3 Diet and experiment design

In a completely randomized experimental design, kittens were randomly assigned to the control group (CON, *n* = 7, two females and five males) and CP group (CP, *n* = 7, two females and five males) based on their initial BW, gender, and litter. This experiment lasted for 56 days. The CON kittens were fed a basal diet, while the CP kittens were fed a basal diet supplemented with 0.1% APS and 0.2% PP. Each kitten was housed separately with ad-libitum access to food.

The diet met the nutrient requirements for raising cats, according to the Association of American Feed Control Officials (AAFCO), and the diet was baked food. The food intake was recorded daily, and BW was recorded on days 0 and 56. The experimental diets were prepared by Qingke Biotechnology Co., Ltd. (Guangzhou, China). The ingredients and analyzed chemical compositions of the basal diet are shown in Table 1.

**Table 1:** Dietary ingredients and analyzed chemical compositions of the basal diet.

Items	CON	CP
Ingredients (as-is basis, %)		
Chicken meal	50.00	50.00
Fish meal	20.00	20.00
Duck meal	12.00	12.00
Chicken liver	5.00	5.00
Krill meal	2.00	2.00
Sweet potato flour	2.00	2.00
Tapioca	2.00	1.70
Salmon oil	1.00	1.00
Whey protein powder	1.00	1.00
Vitamin and mineral	1.00	1.00
Calcium hydrogen phosphate	1.00	1.00
Alfalfa	0.80	0.80
Yolk powder	0.50	0.50
Blueberries	0.50	0.50
Plantain seed	0.50	0.50
Calcium carbonate	0.50	0.50
Glucosamine hydrochloride	0.10	0.10
Yucca powder	0.05	0.05
Chondroitin sulfate	0.05	0.05
*Astragalus* polysaccharide	–	0.1
*Poria cocos* polysaccharide	–	0.2
Chemical compositions		
DM, %	94.70	94.70
OM, % DM basis	92.60	92.60
CP, % DM basis	44.35	44.35
EE, % DM basis	20.60	20.60
CF, % DM basis	2.10	2.10

CON, basal diet group; CP, compound polysaccharide diet group; DM, dry matter; OM, organic matter; CP, crude protein; EE, ether extract; CF, crude fiber.

### 2.4 Serum sample collection and analysis

On day 56, blood samples were collected before feeding. The samples were centrifuged at 3500 rpm at room temperature for 15 min, and the supernatants were collected and stored at −80°C for further analysis. Serum biochemical parameters, including albumin (ALB), globulin (GLO), total protein (TP), albumin/globulin (ALB/GLO), aspartate aminotransferase (AST), alanine transaminase (ALT), amylase (AMY), creatine kinase (CK), creatinine (CRE), urea nitrogen (BUN), urea nitrogen/creatinine (BUN/CRE), glucose (GLU), triglyceride (TG), calcium (Ca), and inorganic phosphorus (IP), were measured by using an automatic biochemical analyzer (SMT-120VP, Chengdu Seamaty Technology Co., Ltd., Chengdu, China). The concentrations of serum immunoglobulin A (IgA), immunoglobulin G (IgG), immunoglobulin M (IgM), interleukin 6 (IL-6), interleukin 8 (IL-8), interleukin 10 (IL-10), interleukin 1β (IL-1β), tumor necrosis factor-*α* (TNF-α), and serum amyloid A (SAA) were detected by using the commercial feline enzyme-linked immunosorbent assay (ELISA) kits (MEIMIAN, Jiangsu Meimian Industrial Co., Ltd., Jiangsu, China). The levels of serum total antioxidant capacity (T-AOC), glutathione peroxidase (GSH-Px), malondialdehyde (MDA), catalase (CAT), and superoxide dismutase (SOD) were measured using commercial kits (Nanjing Jiancheng Bioengineering Institute, Nanjing, China).

### 2.5 16S rRNA high-throughput sequencing analysis

On day 56, fresh feces samples were collected and stored at −80°C for further analysis. Total microbial genomic DNA was extracted from feces using E.Z.N. A.® Stool DNA Kit (D4015, Omega, Inc., USA) following the instructions of the manufacturer. The hypervariable region V3–V4 of the bacterial 16S rRNA gene was amplified with primer pairs 515F (5′-GTGYCAGCMGCCGCGGTAA-3′) and 806R (5′-GGACTACNVGGGTWTCTAAT-3′) by T100 Thermal Cycler PCR thermocycler (BIO-RAD, USA). The Polymerase Chain Reaction (PCR) mixture includes 4 μL of 5 × FastPfu buffer, 2 μL of 2.5 mM dNTPs, 0.8 μL of forward and reverse primers (5 μM), 0.4 μL of FastPfu polymerase, 0.2 μL of BSA, 10 ng of template DNA, and ddH_2_O to a final volume of 20 μL. The PCR amplification cycling was carried out with the initial denaturation at 95°C for 3 min, followed by 27 cycles of denaturing at 95°C for 30 s, annealing at 55°C for 30 s, and extension at 72°C for 45 s, with single extension at 72°C for 10 min, and ending at 10°C. The PCR product was extracted from 2% agarose gel and purified using the PCR Clean-Up Kit (YuHua, Shanghai, China) following the manufacturer’s instructions and quantified using Qubit 4.0 (Thermo Fisher Scientific, USA). Purified amplicons were pooled in equimolar amounts and paired-end sequenced on an Illumina PE300/PE250 platform (Illumina, San Diego, USA) according to the standard protocols by Majorbio Bio-Pharm Technology Co. Ltd. (Shanghai, China).

Raw FASTQ files were de-multiplexed using an in-house Perl script and then quality-filtered by fastp version 0.19.6 and merged by FLASH version 1.2.7. Then, the optimized sequences were clustered into amplicon sequence variants (ASVs) using UPARSE 7.1 with a 97% sequence similarity level. The most abundant sequence from each ASV was selected as a representative sequence. To minimize the effects of sequencing depth on the alpha and beta diversity measures, the number of 16S rRNA gene sequences from each sample was rarefied to 20,000, which still yielded an average Good’s coverage of 99.09%. Based on the ASV information, the subsequent analysis, including the alpha diversity and beta diversity, was conducted using QIIME 2 software.

### 2.6 Serum untargeted metabolomics analysis

The UPLC-Orbitrap-MS/MS analysis, as described in previous research, served as an untargeted metabolomic approach to identify serum metabolic profiles (Xin et al., 2018). Compound Discoverer 2.1 software (Thermo Fisher Scientific, USA) was applied for automated data processing. Metabolites were detected by searching the mzCloud library and mzVault library. Additionally, the potential metabolite markers were filtrated according to the fold change (FC) (FC > 1.2) and t-test (*p* < 0.05). Moreover, these differential metabolites were mapped to the KEGG database for pathway analysis using MetaboAnalyst 5.0.

### 2.7 Statistical analysis

The experimental data were preprocessed by Microsoft Excel 2019. SPSS 26.0 and GraphPad Prism 8.0 software were applied for statistical analysis and graphical presentation. Independent sample Student’s *t*-test was performed for the comparisons between the two groups. Statistical significance was defined as a *p*-value of <0.05. The results were expressed as the mean ± standard error (mean ± SE).

## 3 Results

### 3.1 Effects of CP supplementation on growth performance and serum biochemistry in kittens

As shown in Table 2, there were no significant differences (*p* > 0.05) in IBW, FBW, WG, and DFI between the two groups. The effects of dietary CP supplementation on the serum biochemical parameters in kittens are shown in Table 3. The CP supplementation did not affect the serum biochemistry parameters, including ALB, TP, GLO, ALB/GLO, AST, ALT, AMY, CK, CRE, BUN, BUN/CRE, GLU, TG, Ca, and IP (*p* > 0.05). The serum biochemical parameters of all the kittens were within the reference range.

**Table 2:** Growth performance of kittens during the experiment.

Items	CON	CP	*P*-value
IBW, kg	2.30 ± 0.29	2.48 ± 0.34	0.701
FBW, kg	3.03 ± 0.26	3.31 ± 0.40	0.570
WG, kg	0.73 ± 0.11	0.83 ± 0.12	0.546
DFI, g/d	68.96 ± 2.68	75.51 ± 5.93	0.334

CON, basal diet group; CP, compound polysaccharide diet group; IBW, initial body weight; FBW, final body weight; WG, weight gain; DFI, daily feed intake.

**Table 3:** Effect of CP on serum biochemical parameters.

Items	CON	CP	*p*-value
ALB, g/L	30.31 ± 1.18	30.51 ± 1.29	0.958
TP, g/L	61.50 ± 2.28	61.70 ± 2.37	0.891
GLO, g/L	31.19 ± 1.88	31.17 ± 2.18	0.695
ALB/GLO	0.99 ± 0.06	1.01 ± 0.09	0.269
AST, U/L	22.86 ± 1.78	22.28 ± 1.58	0.779
ALT, U/L	46.14 ± 2.75	49.29 ± 4.83	0.276
AMY, U/L	897.00 ± 74.66	859.14 ± 66.88	0.751
CK, U/L	293.71 ± 97.18	213.00 ± 54.73	0.094
CRE, μmol/L	83.53 ± 5.13	84.74 ± 3.62	0.169
BUN, mmol/L	7.08 ± 0.35	6.73 ± 0.36	0.927
BUN/CRE	87.07 ± 7.37	79.82 ± 4.08	0.154
GLU, mmol/L	3.51 ± 0.26	3.33 ± 0.24	0.834
TG, mmol/L	0.41 ± 0.03	0.45 ± 0.04	0.181
Ca, mmol/L	2.33 ± 0.03	2.31 ± 0.05	0.051
IP, mmol/L	2.03 ± 0.08	2.29 ± 0.09	0.894

CON, basal diet group; CP, compound polysaccharide diet group; ALB, albumin; GLO, globulin; TP, total protein; ALB/GLO, albumin/globulin; AST, aspartate aminotransferase; ALT, alanine transaminase; AMY, amylase; CK, creatine kinase; CRE, creatinine; BUN, urea nitrogen; BUN/CRE, urea nitrogen/creatinine; GLU, glucose; TG, triglyceride; Ca, calcium; IP, inorganic phosphorus.

### 3.2 Effects of CP supplementation on serum antioxidant capacity, inflammatory cytokines, and immunoglobulins in kittens

**Figure 1:**
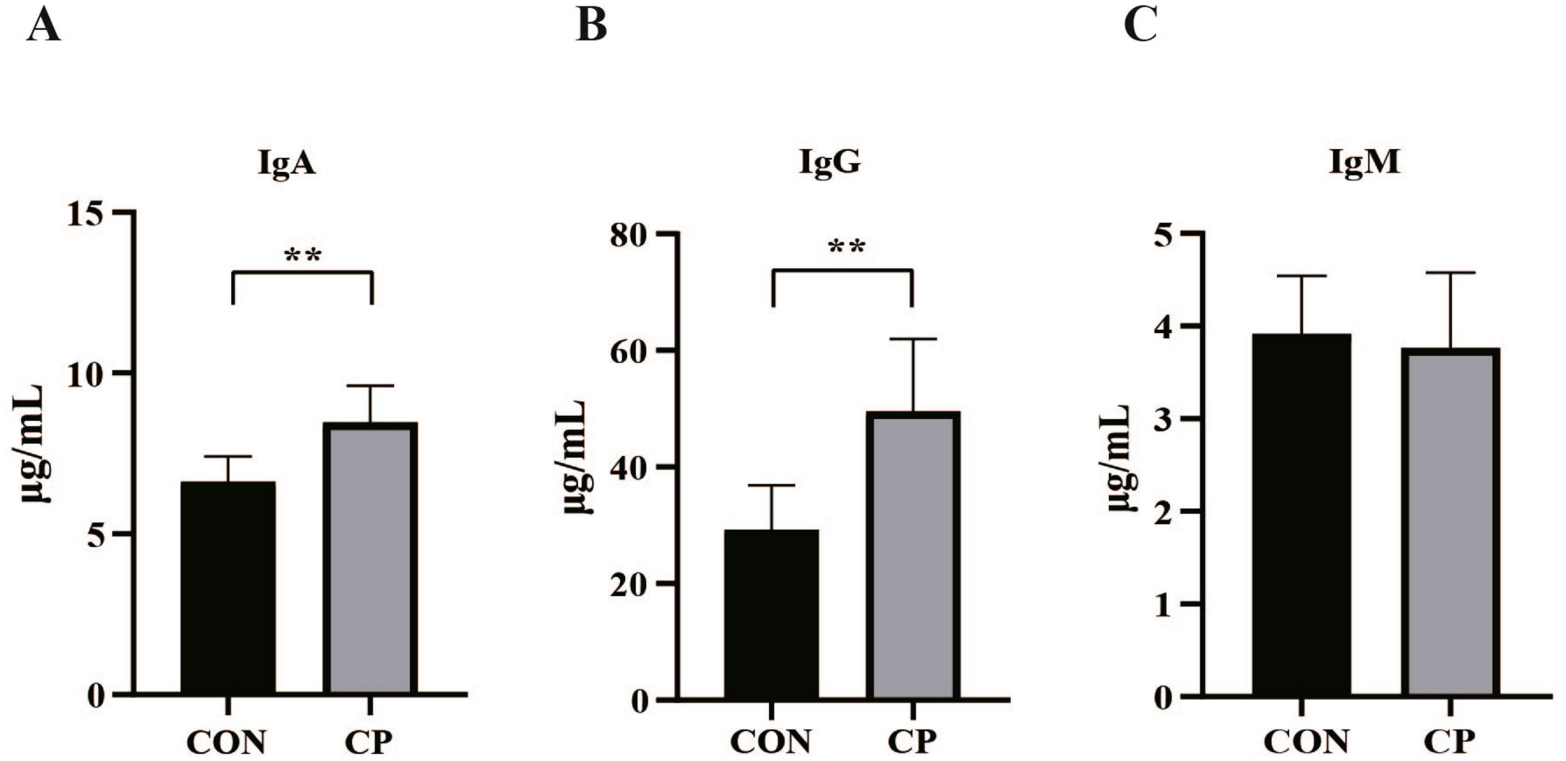
Effects of CP supplementation on the concentrations of serum immunoglobulins in kittens. **(A)** IgA, immunoglobulin A; **(B)** IgG, immunoglobulin G; **(C)** IgM, immunoglobulin M. Data are presented as mean ± SE. The symbol (*) indicates statistically significant differences (**p < 0.01).

**Figure 2:**
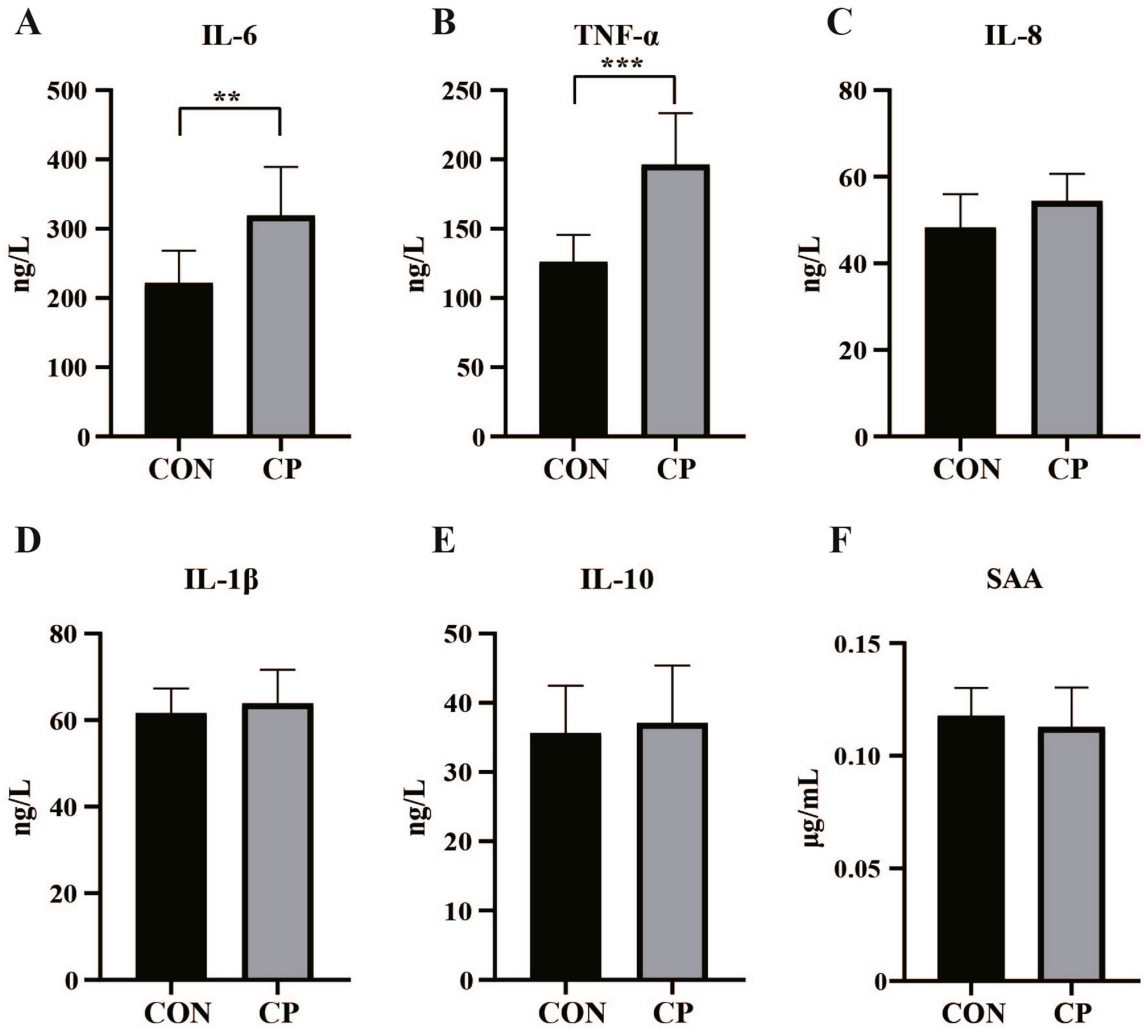
Effects of CP supplementation on the concentrations of serum inflammatory cytokines and markers of inflammation in kittens. **(A)** IL-6, interleukin 6; **(B)** TNF-*α*, tumor necrosis factor-α; **(C)** IL-8, interleukin 8; **(D)** IL-1β, interleukin 1β; **(E)** IL-10, interleukin 10; **(F)** SAA, serum amyloid A. Data are presented as mean ± SE. The symbol (*) indicates statistically significant differences (**p < 0.01 and ***p < 0.001).

The effects of dietary CP supplementation on serum antioxidant capacity are presented in Table 4. There were no differences (*p* > 0.05) in the concentrations of serum antioxidant parameters, including T-AOC, GSH-Px, MDA, CAT, and SOD between the two groups. In addition, CP treatment did not affect the serum IgM, IL-8, IL-1β, and IL-10 (*p* > 0.05, Figures 1C, 2C–E). However, supplementation with CP increased the levels of serum IgA, IgG (*p* < 0.01, Figures 1A,B), IL-6 (*p* < 0.01, Figure 2A), and TNF-*α* (*p* < 0.001, Figure 2B).

**Table 4:** Effects of dietary CP supplementation on serum antioxidant capacity.

Items	CON	CP	*p*-value
T-AOC, mM	0.78 ± 0.04	0.76 ± 0.05	0.478
GSH-Px, pmol/mL	4.50 ± 0.81	4.32 ± 1.08	0.735
MDA, nmol/mL	1.41 ± 0.25	1.28 ± 0.23	0.332
CAT, U/mL	0.28 ± 0.17	0.48 ± 0.29	0.141
SOD, U/mL	9.86 ± 0.79	10.03 ± 0.89	0.715

CON, basal diet group; CP, compound polysaccharide diet group; T-AOC, total antioxidant capacity; GSH-Px, glutathione peroxidase; MDA, malondialdehyde; CAT, catalase; SOD, superoxide dismutase.

### 3.3 Effects of CP supplementation on serum inflammatory markers in kittens

The concentration of serum SAA was not affected by dietary CP supplementation (*p* > 0.05, Figure 2F).

### 3.4 Effects of CP supplementation on serum metabolomics in kittens

**Figure 3:**
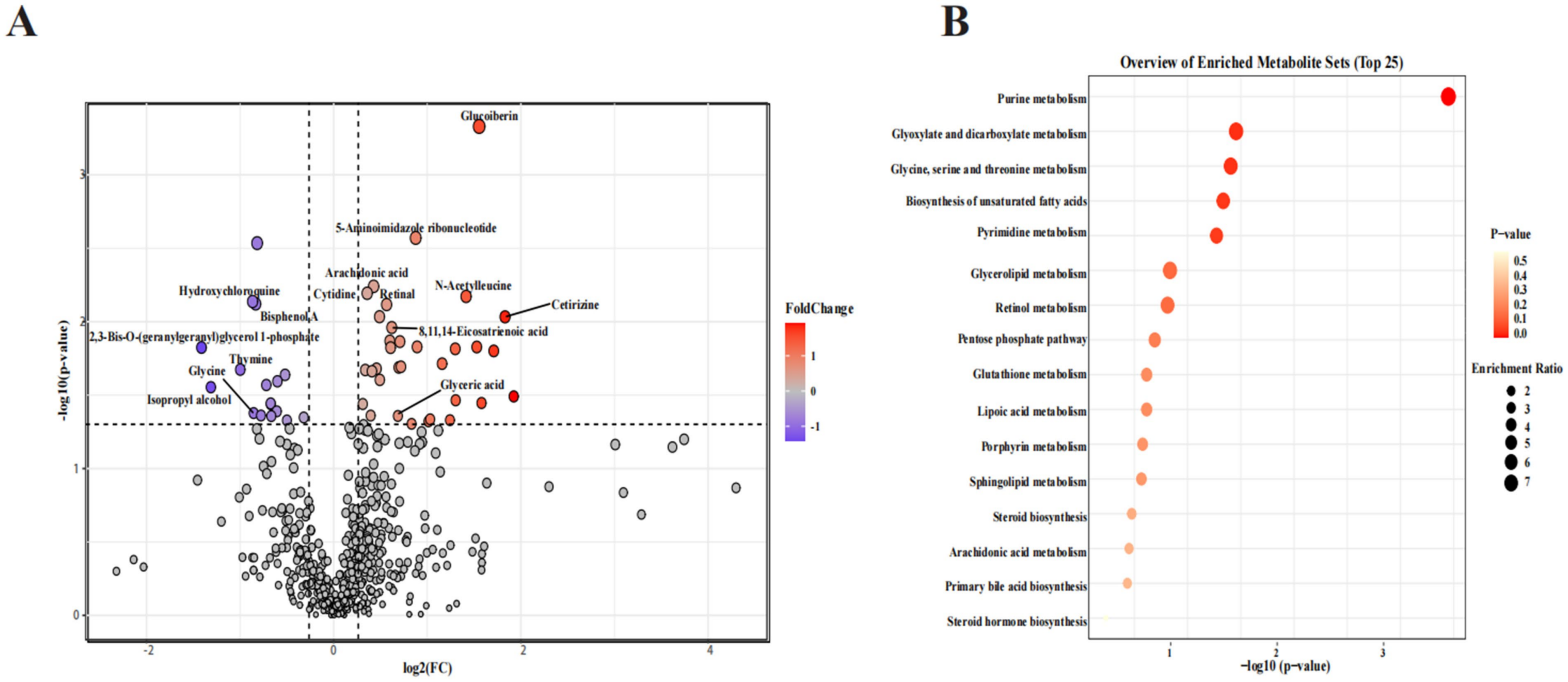
Effects of CP supplementation on the serum metabolome in kittens. **(A)** The volcano plot displayed the differential metabolites between the two groups. **(B)** Bubble chart of the metabolic pathway analysis of differential metabolites between the two groups.

Biomarkers in serum can objectively reflect physiological changes. The UPLC-Orbitrap-MS/MS analysis was used to evaluate the serum metabolic profile. In this study, a total of 759 metabolites were detected in the two groups. Furthermore, potential metabolite markers were screened out using the standard of FC > 1.2 and *p* < 0.05, resulting in 50 differential metabolites between the two groups. Dietary administration of CP upregulated 33 metabolites and downregulated 17 metabolites. The volcano plot displayed that the primary potential metabolite markers were glucoiberin, 5-aminoimidazole ribonucleotide, arachidonic acid (ARA), dihomo-gamma-linolenic acid (DGLA), cytidine, *N*-acetylleucine, hydroxychloroquine, bisphenol A, retinal, cetirizine, 2,3-bis-*O*-(geranylgeranyl)glycerol 1-phosphate, thymine, isopropyl alcohol, glycine, and glyceric acid (Figure 3A).

In addition, as shown in Figure 3B, the KEGG enrichment analysis further explored the effects of dietary CP supplementation on serum metabolic pathways. The results demonstrated that the most dominant pathways were purine metabolism, glyoxylate and dicarboxylate metabolism, glycine, serine, and threonine metabolism, biosynthesis of unsaturated fatty acids, pyrimidine metabolism, glycerolipid metabolism, retinol metabolism, pentose phosphate pathway, and glutathione metabolism.

### 3.5 Effects of CP supplementation on the fecal microbiota in kittens

**Figure 4:**
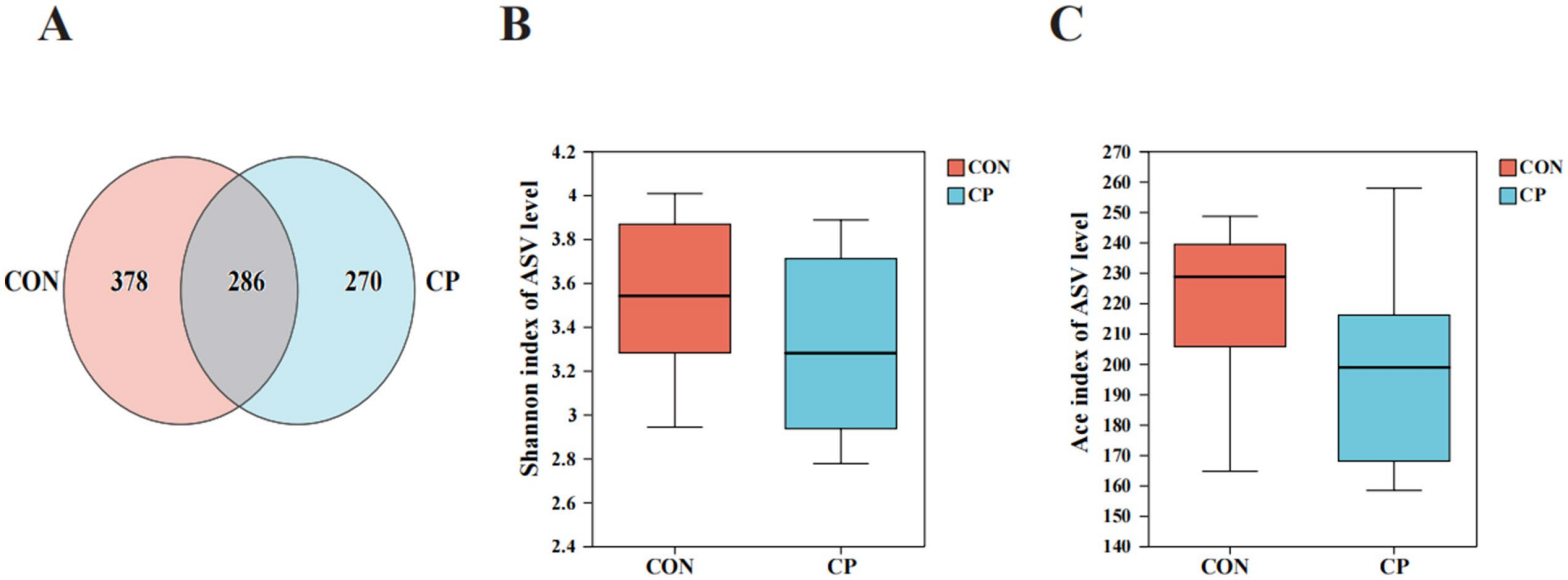
The community diversity of fecal microbiota and the unique and shared ASVs in the two groups. **(A)** Venn diagram of shared ASVs; **(B)** Shannon index of ASV level; **(C)** Ace index of ASV level. CON, control; CP, compound polysaccharide.

**Figure 5:**
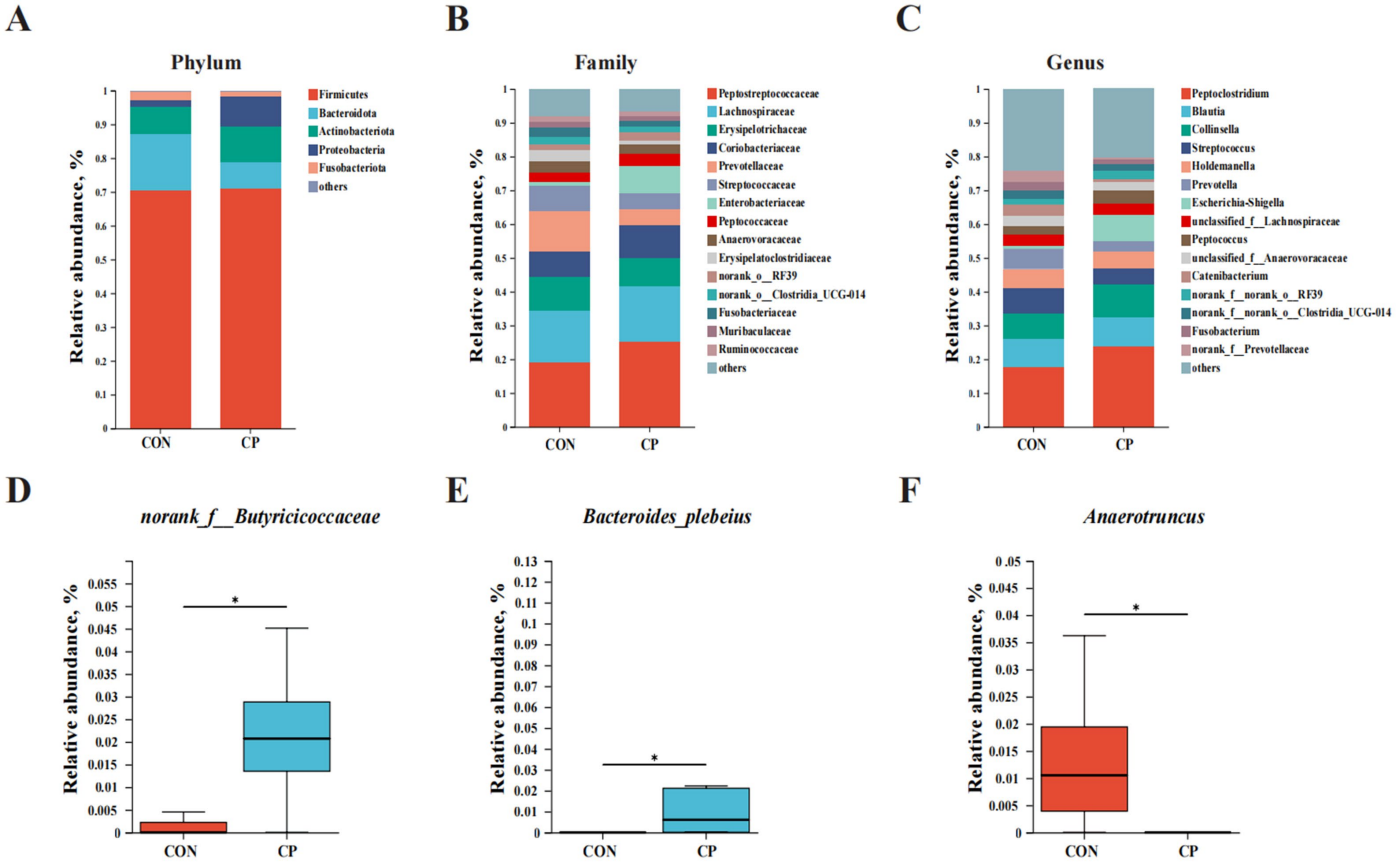
Effects of CP supplementation on intestinal microbiota composition in kittens. Barplot analysis of microbial composition at **(A)** phylum, **(B)** family, and **(C)** genus levels. **(D–F)** The relative abundance of differential bacteria. CON, control; CP, compound polysaccharide. The symbol (*) indicates statistically significant differences (*p < 0.05).

The effects of dietary CP supplementation on fecal microbiota are illustrated in Figures 4, 5. The Venn diagram displayed 286 shared ASVs between the two groups, with 664 ASVs in the CON group and 556 ASVs in the CP group, respectively (Figure 4A). No differences (*p* > 0.05) in the Shannon diversity index and the Ace index were observed between the CON and the CP groups (Figures 4B,C).

At the phylum level, the dominant bacteria included *Firmicutes*, *Bacteroidota*, *Actinobacteriota*, *Proteobacteria,* and *Fusobacteriota* (Figure 5A). At the family level, the predominant bacteria included *Peptostreptococcaceae*, *Lachnospiraceae*, *Erysipelotrichaceae*, *Coriobacteriaceae*, *Prevotellaceae*, *Streptococcaceae*, *Enterobacteriaceae*, and *Peptococcaceae* (Figure 5B). At the genus level, the dominant bacteria consisted of *Peptoclostridium*, *Blautia*, *Collinsella, Streptococcus*, *Holdemanella*, *Prevotella*, *Escherichia-Shigella*, *unclassified_f__Lachnospiraceae*, and *Peptococcus* (Figure 5C). Moreover, the relative abundance of *norank_f__Butyricicoccaceae* and *Bacteroides plebeius* was enriched in the CP group, while the relative abundance of *Anaerotruncus* was enriched in the CON group (*p <* 0.05, Figures 5D–F).

## 4 Discussion

### 4.1 Dietary supplementation with CP increased the concentrations of serum IgA, IgG, IL-6, and TNF-*α*

Macrophages play an essential role in both adaptive and innate immunity as the antigen-presenting cells, secreting pro-inflammatory cytokines, and cleaning foreign antigens (Deng et al., 2023). Previous studies have found that PP can increase the production of pro-inflammatory cytokines IL-6, TNF-*α*, and IL-12 by inducing M1 macrophage polarization (Hu et al., 2023; Zhao R. et al., 2023). The concentrations of IL-6, TNF-*α*, and IL-1β were significantly enhanced after PP treatment, with immune regulation attributed to the activation of the Ca2+/PKC/p38/NF-κB signaling pathway (Pu et al., 2019). In addition, cellular tests indicated that PP can combine with mannose receptors in macrophages, enhancing the levels of TNF-α and mRNA expression in cells (Zhang et al., 2024).

Furthermore, research has proven the immunomodulatory effect of APS by enhancing the activities of macrophages, natural killer cells, T lymphocytes, and B lymphocytes, leading to the secretion of cytokines (Li C. et al., 2022; Zhao R. et al., 2023). Dietary supplementation with APS has been shown to increase the serum TNF-*α*, IL-1β, and IgA in weaned piglets by activating the TLR4-mediated MyD88-dependent signaling pathway (Wang K. et al., 2020). Treatment with APS could resist the infection of the pathogen *Brucella* and alleviate the symptoms of mice with brucellosis by activating the macrophages and upregulating the production of TNF-*α*, IL-12, and IFN-*γ* (Shi et al., 2019). Furthermore, supplementation with APS effectively increased serum concentrations of IL-6, TNF-*α*, and IFN-γ in weaning rex rabbits (Sun et al., 2016).

The above results demonstrate that APS and PP display immunostimulatory functions by enhancing the secretions of pro-inflammatory cytokines and immunoglobulins. Similar results were observed in our study, where CP supplementation increased the concentrations of serum IL-6, TNF-α, IgA, and IgG in kittens.

Previous studies have indicated that IL-6 and TNF-*α* were pro-inflammatory cytokines, which mediate in many inflammatory diseases and contribute to developing autoimmune disorders and cancer (Waldner and Neurath, 2014). However, a number of studies have also demonstrated that IL-6 is a multifunctional cytokine that can induce B cells into mature plasma cells, thus improving antibody production (Urashima et al., 1996). Moreover, IL-6 is involved in the proliferation and differentiation of T cells, and mice lacking IL-6 often show deficiencies in T cell effector function and memory recall (Kopf et al., 1994; Twohig et al., 2019; Urashima et al., 1996). Furthermore, IL-6 plays an essential role in protecting mice from *Escherichia coli* infection by efficiently inducing neutrophils in the bloodstream (DALRYMPLE et al., 1996).

TNF-*α* is indispensable for adaptive immune responses as it is necessary for developing lymphoid organs and can activate lymphocytes, thus enhancing the secretion of antibodies and cytokines to eliminate infection (Li et al., 2001). For example, TNF-α is essential for mice to eliminate hepatocytes infected by hepatitis B virus in the immune response to hepatitis B virus. TNF-α also plays an important role in regulating cytokines derived from astrocytes, which are involved in responding to microbial infections (Phulwani et al., 2008). In brief, IL-6 and TNF-*α*, crucial immunomodulators, play an important role in resisting foreign pathogens and injuries by mediating the activation of lymphocytes and dendritic cells and inducing the production of antibodies.

Generally, the systemic inflammation response can improve immunity and resistance to infection, benefiting the host. However, an excessive pro-inflammatory response harms the whole health (Bistrian, 2004). A moderate increase in IL-6 and TNF-α can enhance the overall immunity of kittens. To confirm that the immune enhancement of CP supplementation did not lead to generalized inflammation, we measured the concentration of serum SAA. SAA, one of the serum acute phase proteins (APPs), serves as an inflammatory marker for cats. In addition, SAA is a major positive APP, which is more sensitive than white blood cell counts for early detection of inflammation (Rossi, 2023; Tuna and Ulutas, 2022). Under the stimulation of inflammatory cytokines, such as IL-6 and TNF-*α*, SAA is synthesized by hepatocytes in the liver and released into the circulating blood, resulting in a systemic inflammatory response (Ishioka and Hayakawa, 2019). The concentration of serum SAA increases during acute or chronic inflammation (Waugh et al., 2022). In this study, no difference in the concentration of serum SAA was found after CP treatment. Moreover, the concentrations of serum SAA in both groups fell within the normal reference range, as reported in previous research (Waugh et al., 2022). Thus, it is clear that the immune enhancement effect of CP treatment did not cause generalized inflammation.

However, there are many studies demonstrating that APS and PP display an anti-inflammatory effect through a decrease in the production and secretion of inflammatory cytokines, such as IL-1β, IL-6, and TNF-*α* (He et al., 2022; Ming et al., 2022; Ng et al., 2024; Wu et al., 2022), which are contrary to our results. It is worth noting that the anti-inflammatory effects of APS and PP are displayed in the inflammation model or hosts experiencing inflammation. Conversely, the pro-inflammatory effect can be observed in the immunosuppression model or hosts under immunosuppressive conditions. Therefore, the effects of supplementation with CP depend on the immune status of the host. In this study, kittens were more susceptible to diseases because of the reduced maternal antibodies, immature immune system, and challenging events in their early life; thus, dietary supplementation of CP enhanced the levels of serum IL-6, TNF-*α*, IgA, and IgG, which contributed to improving their overall immunity of the kittens.

### 4.2 The untargeted serum metabolomics analysis reveals the metabolic mechanism of CP supplementation in kittens

The untargeted serum metabolomics analysis was applied to uncover the potential mechanism of CP supplementation in regulating inflammatory responses in kittens. In this study, serum metabolomic analysis revealed that the metabolic markers significantly altered by CP supplementation were ARA, DGLA, glycine, and glyceric acid. Moreover, further pathway analysis revealed the metabolic pathways regulated by the above differential metabolites, which play a role in the immunomodulatory effects of kittens.

Previous studies indicated that glycine plays an important part in regulating inflammatory cytokines by inhibiting the activities of macrophages, monocytes, and T lymphocytes. Furthermore, the level of serum glycine is negatively correlated with the level of TNF-*α* (Gu et al., 2012; Li et al., 2001; Yao et al., 2015). In our study, pathway analysis indicated that glycine and glyceric acid were the enriched metabolites in the glycine, serine, and threonine metabolism and glyoxylate and dicarboxylate metabolism pathways. In addition, supplementation with CP decreased the level of glycine and increased the concentration of TNF-*α* in serum, which is consistent with the results in the above studies.

DGLA and ARA are both omega-6 polyunsaturated fatty acids, and the balance between DGLA and ARA is important in regulating inflammatory processes in the body (Mustonen and Nieminen, 2023). Previous research has demonstrated that DGLA, serving as a precursor for the synthesis of ARA, can be metabolized via delta-5 desaturase reaction to ARA in cats (Trevizan et al., 2012). ARA plays an essential role in inflammatory processes by stimulating the production of pro-inflammatory cytokines, such as TNF-α, IL-6, and IL-1β (Monmai et al., 2020). Previous studies have proved that ARA is the main precursor of eicosanoids, such as prostaglandins, leukotrienes, thromboxanes, lipoxins, hepoxilins, hydroxyeicosatetraenoic acids, and epoxyeicosatrienoic acids, which are mediators of inflammation (Calder, 2008; Ming Luo, 2006; Panigrahy et al., 2010). Specifically, ARA is present in the membranes of most mammalian cells and can be released from the cell membrane through mobilization by various phospholipase enzymes; thus, free ARA can participate in the synthesis of eicosanoids (Ming Luo, 2006; Calder, 2008; Panigrahy et al., 2010). For example, ARA can be converted to hydroxyeicosatetraenoic acids and epoxyeicosatrienoic acids by cytochrome P450 enzymes (Panigrahy et al., 2010). These ARA-derived eicosanoids were found to modulate the production of inflammatory cytokines (Esser-von Bieren, 2017). For example, the concentrations of TNF-α and IL-6 in cell-free supernatants increased after the monocytes were exposed to leukotriene B4 (Rola-Pleszczynski and Stankova, 1992). The above research indicated that the biosynthesis of unsaturated fatty acids and related metabolites plays a crucial role in immune regulation. Our results revealed that CP supplementation upregulated the concentrations of ARA and DGLA in serum, with consequent effects on the biosynthesis of unsaturated fatty acids. In our study, supplementation with CP increased the concentrations of serum DGLA, ARA, TNF-α, and IL-6 and decreased the level of glycine, suggesting that DGLA, ARA, and glycine may serve as mediators for CP in regulating immune function in kittens.

### 4.3 Dietary supplementation with CP did not generate a phylogenetic change in the fecal bacterial composition of kittens

The results showed that the predominant phyla were *Firmicutes*, *Bacteroidota*, *Actinobacteriota*, *Proteobacteria,* and *Fusobacteriota*, which were consistent with fecal microbial composition in healthy cats as described in previous research (Bierlein et al., 2021; Garcia-Mazcorro et al., 2011). Furthermore, there were no differences in the relative abundance of these predominant phyla between the two groups, suggesting that the supplementation of CP did not alter the predominant phyla in kittens. However, the supplementation with CP increased the relative abundance of *norank_f__Butyricicoccaceae* and *Bacteroides plebeius* (*p* < 0.05) while decreasing the abundance of *Anaerotruncus* (*p* < 0.05).

The Butyricicoccaceae family is a group of essential butyrate-producing bacteria. Previous studies have indicated the beneficial effects of *Butyricicoccaceae* on improving the intestinal health of the host (Ameer et al., 2023; Guo et al., 2022; Yu et al., 2023). The *Bacteroides plebeius* served as probiotics, and it was the producer of propionate in the canine intestine (Kaelle et al., 2023; Sun et al., 2023). Moreover, studies in cats have indicated that *Bacteroides plebeius* are significantly abundant in healthy cats compared to those with chronic enteropathy (Li X. et al., 2022; Marsilio et al., 2019). Previous research has indicated that *Bacteroides plebeius* can enhance the barrier function of the intestinal mucosa by modulating the gut microbiome, reducing protein consumption in rats with chronic kidney disease, and increasing the abundance of probiotics while minimizing damage to the intestinal mucosal barrier (Pei et al., 2022).

*Anaerotruncus* is an opportunistic pathogen that has been linked to inflammatory bowel disease as it can induce the production of pro-inflammatory cytokines and cause immune disorder (Liu et al., 2021). A previous study demonstrated that Fuzi polysaccharide treatment significantly decreased the relative abundance of *Anaerotruncus,* and the mRNA expression of IL-6 was negatively correlated with the relative abundance of *Anaerotruncus* in the immunosuppressive mouse model, which is consistent with our findings (Tu et al., 2023). In a word, administration with CP caused several alterations in the relative abundance of certain bacteria groups, leading to some beneficial effects in kittens. However, CP treatment did not generate a phylogenetic change in the fecal bacterial composition in kittens.

To the best of our knowledge, this is the first time to evaluate the effect of compound polysaccharides on immunity, antioxidant capacity, gut microbiota, and serum metabolome in kittens. However, it is important to note that there are some limitations to this study. A major limitation of our study is that while the supplementation of CP did generate some advantageous effects on kittens, we cannot determine the individual contributions of each polysaccharide in CP to the observed results. In addition, the time-dependent effects of CP administration were not assessed during the study. Clearly, more research is needed to investigate the respective effects of APS and PP to evaluate their potential beneficial properties in kittens. Moreover, further studies should consider increasing the sampling frequency to evaluate the effect of time and the interaction between diet and time of CP treatment in kittens.

## 5 Conclusion

The serum biochemistry parameters were within the normal range for kittens, and all were under normal physiological conditions. Supplementation with CP increased the concentrations of serum immunoglobulins and inflammatory cytokines, but it did not affect the level of serum SAA. These results indicate that dietary supplementation with CP has the potential to support the immunity of kittens and increase the relative abundance of beneficial gut microbiota. It is a favorable immunonutritional supplement in pet food.

## Data Availability

The original contributions presented in the study are publicly available. This data can be found here: NCBI, accession PRJNA1226968.
